# Plasma-derived exosomal miRNA as potential biomarker for diagnosis and prognosis of vector-borne diseases: A review

**DOI:** 10.3389/fmicb.2023.1097173

**Published:** 2023-04-13

**Authors:** Gokulakannan Venkatesan, Wan Suriana Wan Ab Rahman, Wan Nazatul Shima Shahidan, Salfarina Iberahim, Alwi bin Muhd Besari@Hashim

**Affiliations:** ^1^School of Dental Sciences, Universiti Sains Malaysia, Kubang Kerian, Kelantan, Malaysia; ^2^Department of Haematology, School of Medical Sciences, Universiti Sains Malaysia, Kubang Kerian, Kelantan, Malaysia; ^3^Department of Internal Medicine, School of Medical Sciences, Universiti Sains Malaysia, Kubang Kerian, Kelantan, Malaysia

**Keywords:** vector-borne diseases, exosomes, plasma, miRNA—microRNA, biomarker

## Abstract

Early disease diagnosis is critical for better management and treatment outcome of patients. Therefore, diagnostic methods should ideally be accurate, consistent, easy to perform at low cost and preferably non-invasive. In recent years, various biomarkers have been studied for the detection of cardiovascular diseases, cerebrovascular diseases, infectious diseases, diabetes mellitus and malignancies. Exosomal microRNA (miRNA) are small non-coding RNA molecules that influence gene expression after transcription. Previous studies have shown that these types of miRNAs can potentially be used as biomarkers for cancers of the breast and colon, as well as diffuse large B-cell lymphoma. It may also be used to indicate viral and bacterial infections, such as the human immunodeficiency virus (HIV), tuberculosis and hepatitis. However, its use in the diagnosis of vector-borne diseases is rather limited. Therefore, this review aims to introduce several miRNAs derived from exosomal plasma that may potentially serve as a disease biomarker due to the body’s immune response, with special focus on the early detection of vector-borne diseases.

## Introduction

Extracellular vesicles (EVs) are a heterogeneous group of membrane vesicles secreted by nearly all cell types in normal physiology, and the secretion of their content is influenced by the cells’ current pathophysiological state (Yáñez-Mó et al., 2015). They are classified based on their generation mechanism, including ectosomes, apoptotic bodies and exosomes (Martelli et al., 2018). Ectosomes, known as microvesicles, typically range from 200 to 2,000 nm in size and is generated through outward buddings from the plasma membrane (Wu et al., 2021). Microvesicles mainly contain cytosolic and plasma membrane-associated proteins, such as tetraspanins. They also carry cytoskeletal proteins, heat-shock proteins, integrins and post-translationally modified proteins that have undergone glycosylation and phosphorylation (Doyle and Wang, 2019). The discovery of new proteins in microvesicles have spurred global interest in fully understanding their contents for diagnostic and therapeutic potential. In malaria infection caused by *Plasmodium vivax*, a rise in the level of plasma-derived microvesicles has been observed in the blood of patients, which potentially allows disease detection at an earlier stage (Campos et al., 2010). Apoptotic bodies, meanwhile, are vesicles with a size ranging from 500 nm to 2 μm, and are discharged by dying cells into the extracellular space. They contain broken down cellular particles, such as intact organelles, chromatin and small amounts of glycosylated proteins (Borges et al., 2013). A recent study has demonstrated that these vesicles may be used as a non-invasive method to track apoptosis in patients with cerebrovascular and neurodegenerative diseases for prognostic purposes (Serrano-Heras et al., 2020).

Exosomes are nanometer-sized (50–150 nm) intraluminal vesicles (ILV) of multivesicular bodies (MVB) secreted by various cells, including the microglia (Mashouri et al., 2019; Fan et al., 2022). They have double membrane structure (Saadatpour et al., 2016) that originates from endocytic compartments and are matured in the MVB of late endosomes (Statello et al., 2018). MVBs can fuse with the plasma membrane, leading to the release of ILVs into the extracellular space as exosomes (Colombo et al., 2014). Exosomes are surrounded by a lipid bilayer, carrying a variety of biomolecules, such as glycans, proteins, metabolites, lipids, DNA, and RNA, including miRNA (Chung et al., 2020). They can potentially serve as an important medium for cell-to-cell or cell-to-internal environment communication (Jeppesen et al., 2019), and the biomolecules may alter physiological responses by mediating short- and long-distance inter-organ communication (Fatima and Nawaz, 2017). Exosomes are less immunogenic, non-cytotoxic and non-mutagenic to their target cells compared with other gene delivery vehicles (O’Loughlin et al., 2012). Depending on the molecules they carry, exosomes can perform a variety of roles, such as encouraging cell proliferation, migration, lowering oxidative stress and preventing apoptosis, besides mediating immune responses like cytokine secretion and inhibition or stimulation of inflammasomes (Noonin and Thongboonkerd, 2021). Emerging findings have suggested that exosomes may play a key role in mediating infection and inflammatory processes (Xie et al., 2022).

Infection by a vector-borne disease will trigger the effects of a host-parasite interaction, which facilitates the parasite’s long-term survival in the host body (Podolska and Nadolna, 2014). The host-parasite interaction can also trigger the host immune response and enhance pathogen diffusion (Cox et al., 2012). As parasites interact with host cells through excretory-secretory channels, the exosomes within excretory-secretory products will naturally serve as a route for successfully mediating the parasite’s contact for host manipulation. It also makes sense that the host would utilize this pathway as a defense mechanism (Nawaz et al., 2019).

Recently, exosomes have gained much research attention in biomarker development as they contain specific proteins, microRNAs (miRNAs) and signaling molecules that are associated with disease progression (Hoshino et al., 2015). Exosome miRNA is highly accessible and stable, thus raising its potential use in precision medicine (Muinelo-Romay et al., 2018). However, studies on the role of exosomes in vector-borne diseases are nonetheless limited by inefficient separation techniques, a lack of distinctive biomarkers, and challenges in obtaining high-resolution imaging methods. Exosomes contain identical protein and RNA profiles to the cells that produce them. However, very few distinct cell-specific proteins and miRNAs exist (Li et al., 2017). Furthermore, exosomes from pathogen-infected cells are heterogeneous, and their concentration exists in a different range than the standard level (Reyes-Ruiz et al., 2019). In this review, we collated and updated the role of exosomal plasma miRNA in various diseases, and then examined the scope of miRNA-derived from plasma exosomes as a reliable diagnostic tool for vector-borne diseases.

## Methodology and delimitation

Article search and data collection were performed on PubMed, Google Scholar, and World Health Organization (WHO) databases. It included peer-reviewed articles with original data from 2010 to 2022. Non-peer-reviewed articles, conference abstracts, letters, articles without full-text were excluded. All articles were collected and compiled using the Mendeley application to eliminate duplicates. A PubMed database search using the keywords “extracellular vesicles and vector bore diseases” resulted in a total of 225 articles. After subsequently applying the keywords “exosomes and vector-borne disease,” a total of 115 articles were produced. As the search was narrowed down with the keywords “miRNA biomarker and vector bore diseases,” the articles were further reduced to 79. Finally, 31 articles remained when the second parameter “plasma miRNA and vector-borne diseases” was added. The articles were manually divided into two groups: “original articles” and “others” (not related to topic, reviews, reports, editorials, commentaries, etc.). The original articles addressing plasma exosome miRNA and vector-borne diseases became the focus of the study and were used as material in this review.

## Plasma-derived exosomes

Exosomes are critical paracrine mediators that may be detected in bodily fluids, including plasma (Carrozzo et al., 2021). The content of human plasma exosomes have been linked to a variety of physiological and pathological conditions (Yáñez-Mó et al., 2015). Such exosomes may be isolated using ultracentrifugation, polymer-based precipitation kits, size exclusion chromatography, immunoaffinity, and ultrafiltration (Kalra et al., 2013). Ultracentrifugation and polymer-based precipitation kits are the most popular methods, but the former has several limitations, including low exosome concentration and considerable albumin contamination (Baranyai et al., 2015). These may be resolved by changing the isolation parameters, such as viscosity, sedimentation distance and temperature, or by prolonging the ultracentrifugation time to increase yield (Koh et al., 2018). The presence of exosomes may be characterized using complex methods, such as nanoparticle tracking analysis, electrophoretic light scattering, transmission electron microscopy and EXOCET colorimetric assay, or simple protein quantification like Western blotting (Martins et al., 2018). In blood samples, plasma exosomes have a higher concentration of miRNA compared with serum, and this may serve as a better sample for blood-based proteomics research of exosomes (Cao et al., 2019). Plasma cells may be more protected from degradation and this enables more accurate distribution of target cells, providing a distinctive perspective into pertinent diseases, or defining novel diagnostic and prognostic potential for different disorders (Ludwig et al., 2017). Hence, plasma-derived exosomes are a clinically valuable biomarker to assess the immune system of patients because its molecular profiling and protein monitoring may show the degree of immunological suppression before and during treatment (Ludwig et al., 2017).

## Plasma-derived exosomal miRNA in diagnosing diseases

MiRNA are naturally occurring short non-coding RNA molecules with a length of about 21–25 nucleotides (Ha and Kim, 2014). MiRNA from exosomes may affect the body’s physiological modulation and disease progression (Liu et al., 2020). Depending on disease condition, the level of miRNA in bodily fluids will be up- or down-regulated (Abdellatif, 2012). The miRNA level in blood, for example, is affected by various factors and will differ from sample to sample (e.g., plasma, serum, and other fluids) (Blondal et al., 2013). Hemolysis can influence the expression of miRNA in serum, but plasma-derived miRNA is unaffected (Foye et al., 2017). MiRNA in the exosomal plasma are in more stable form than any other bodily fluids because they are protected from endogenous RNase degradation (Köberle et al., 2013). This makes them useful in the diagnosis of infectious diseases, besides diseases of the central nervous system and respiratory systems, including malignancy (Alipoor et al., 2016).

During the early stage of human immunodeficiency virus-1 (HIV-1) infection, changes in exosomal miR-155 expression play a role in the development of an effective antiviral effector response by CD8 T cells and at the same time increase the number of regulatory T cells (Lind et al., 2013). Thus, exosomal miRNA involved in the regulation of immune and inflammatory responses, and also serve as an excellent companion in the diagnosis of HIV infection (Hubert et al., 2015). Tuberculosis is another infectious disease that is currently screened by microscopic examination and confirmed using genotypic assays (line probe assay, cartridge-based nucleic acid amplification test, or loop-mediated isothermal amplification), besides the gold standard culture methods (liquid culture media) (Chopra and Singh, 2020). Still, there is research on finding a reproducible, efficient, cost-effective tool with minimal infrastructure requirements for this infectious disease. Recently, upregulated miRNA-185-5p expression in exosome plasma has been identified in tuberculosis (Kaushik et al., 2021). In hepatitis C (HCV), immunomodulatory miRNA enrichment exosomes are found to be associated with their inhibitory activity on innate immune cell function. This may provide valuable biomarkers to monitor immune response recovery (Santangelo et al., 2018).

Most recently, reverse-transcription polymerase chain reaction (RT-PCR) has been mainly used to confirm COVID-19 infection. However, false-negatives may occur in a small percentage of tests due to a low viral load in patient samples (Anand et al., 2021). The use of exosomes as a diagnostic tool for COVID-19 has yet to be widely studied. Therefore, there is an immediate need to study and develop an alternative method that is more sensitive, specific and easy to apply. The study of the exosomal cargo may provide key information with respect to differential secretion of miRNA in SARS-CoV 2 infected cells compared with uninfected cells. Table 1 summarizes the exosomal plasma-derived miRNA biomarkers for diagnostic applications in various diseases. The preceding studies support the notion that plasma-derived exosomal miRNAs play important roles in diseases as a promising biomarker and provide knowledge for developing a new diagnostic tool for the diseases.

**TABLE 1:** Plasma-derived exosomal miRNA expression in various diseases.

Disease	Exosome characterization	miRNA profiling	miRNA	References
Breast cancer	Electron microscopy, nanoparticle tracking analysis and Western blot	Next-generation small RNA sequencing	miR-21 and miR-1246	Hannafon et al., 2016
Colon cancer	Scanning electron microscopy	Real-time qRT-PCR	miR-125a-3p	Wang et al., 2017
Diffuse large B-cell lymphoma	Scanning transmission electron microscopy and scanning electron microscopy	miRNA expression microarray	miR-3960, miR-6089 and miR-939-5p	Caner et al., 2021
Pancreatic ductal adenocarcinoma	Transmission electron microscopy	Microarray analysis	microRNA-451a	Takahasi et al., 2018
Papillary thyroid carcinoma	Transmission electron microscopy, Western blot, flowcytometry for nanoparticle analysis	RNA sequencing	miR-16-2-3p, miR-223-5p, miR-101-3p and miR-34c-5p	Liang et al., 2020
Rheumatoid arthritis	Electron microscopy analysis	Microarray analysis	miRNA-204-5p	Wu et al., 2022
Myocardial infraction	Transmission electron microscopy	Microarray analysis	miRNA-183	Zhao et al., 2019
Type 1 diabetes mellitus	Nanoparticle tracking analysis and transmission electron microscopy	Nano-string human v2 miRNA microarray	miR-631	Garcia-Contreras et al., 2017
HIV-1	Western blot, electron microscopy and dynamic light scattering		miR-155 and miR-223	Hubert et al., 2015
Tuberculosis		Next-generation small RNA sequencing	miRNA-185-5p	Kaushik et al., 2021
Alzheimer disease	Western blot	Illumina HiSeq2500	miR-141-3p	Lugli et al., 2015

## Plasma-derived exosomal miRNA in gene regulation

From DNA sequences, miRNAs are translated into precursor miRNAs (pre-miRNAs), mature miRNAs and primary miRNAs (pri-miRNAs). MiRNAs regulate protein translation by binding to the 3’ untranslated regions of mRNA template (MacFarlane and Murphy, 2010). However, it has been reported that they can also interact with other regions, including the 5’ UTR coding sequence and gene promoters (Broughton et al., 2016). These miRNAs play a crucial role in influencing molecular and cellular processes in cancer and inflammation (O’Connell et al., 2012).

Enterotoxigenic *Bacteroides Fragilis* (ETBF) is an enterotoxin-producing bacterium that is strongly associated with inflammatory bowel disease (IBD), colitis-associated colorectal cancer and colorectal cancer (CRC) as it has been observed to change the mucosal immune response and induce epithelial cell changes (Zamani et al., 2020). A recent study on ETBF shows that the exosomal down- regulated miR-149-3p derived from ETBF-treated cells facilitated T-helper type 17 cell differentiation and promoted the PHF5A (Plant homeodomain finger protein 5A) mediated RNA alternative splicing of the KAT2A gene in CRC cells. Thus, the ETBF/miR-149-3p pathway showed a potential solution for treatment of patients with intestinal inflammation and CRC with a high amount of ETBF (Cao et al., 2021). Plasma-derived exosomal miR-216a-5p may also inhibit rTp17-induced inflammatory cytokine production and the TLR4-MYD88 signaling pathway in syphilis infection, suggesting a possible therapeutic target for inflammation caused by the sexually transmitted disease (Peng et al., 2019).

Medulloblastoma (MB) is a malignant brain tumor that commonly affects children. Exosomal miR-101-3p and miR-423-5p from plasma of MB patients have been identified to act as tumor suppressors by directly targeting *FOXP4* and *EZH2* oncogenes (Xue et al., 2022). This shows that plasma-derived exosomal miRNA can control the expression of oncogenes, making them a potential therapeutic agent. An interesting study on long-term exercise-derived plasma exosome found that miR-342-5p may prevent ischemic heart disease by suppressing the level of Caspase 9 and JNK2, thus reducing hypoxia/reoxygenation-induced cardiomyocyte apoptosis; moreover, it increased survival signaling (p-Akt) by targeting the phosphatase gene *Ppm1f* (Hou et al., 2019). These studies provided the information needed to understand the role plasma-derived exosomal miRNA as a therapeutic agent by controlling the expression of genes that cause various diseases.

## Role of exosomes in vector-borne diseases

**FIGURE 1:**
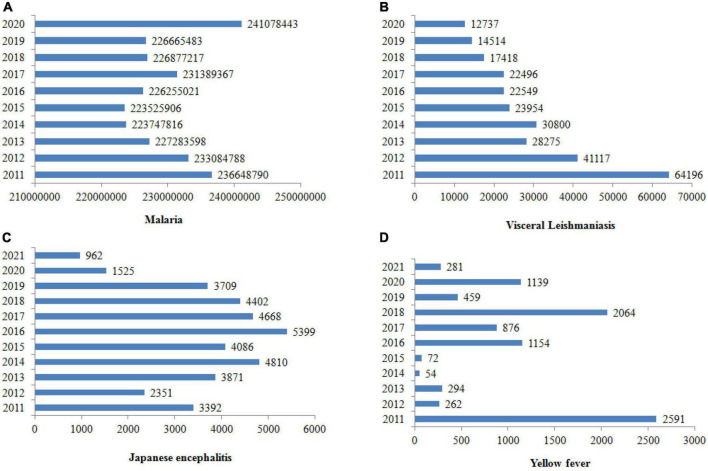
Cases of vector-borne diseases worldwide from 2011 to 2020. The prevalence of malaria is rising, but leishmaniasis, Japanese encephalitis and yellow fever seem to be declining (World Health Organization, 2020a,b, 2021, 2022d).

Vector-borne diseases are infections caused by pathogens that are usually transmitted by arthropods, such as mosquitoes, triatomine bugs, black flies, tsetse flies, sand flies, lice and ticks (Golding et al., 2015). However, they may also include other carriers such water snails, which serve as a reservoir for *Schistosomiasis*. In the case of insects, they include dengue, Chagas disease, Japanese encephalitis, leishmaniasis, lymphatic filariasis, malaria, and yellow fever, which all together threaten more than 80% of the world’s population. They are inordinately distributed in the tropics and subtropics (Chala and Hamde, 2021), and the World Health Organization (WHO) estimated that they account for more than 700,000 deaths annually (World Health Organization, 2022a). Figure 1 shows the number of vector-borne cases reported worldwide from 2011 to 2020. Table 2 summarizes the laboratory method for vector-borne disease detection in the host and its vectors.

**TABLE 2:** Diagnostic methods for vector-borne disease detection in the host and their vectors.

Vector-borne disease	Pathogen	Vector	WHO guideline on pathogen detection	Method of detection in vector	Exosome detection in vector	References
Dengue	DENV	*Aedes aegypti*	NS1 antigen, IgG and IgM detection using ELISA, rapid point-of-care tests and RT- PCR	Reverse transcription-polymerase chain reaction test (RT-PCR)	Cryo-electron microscopy	Vora et al., 2018; Ali et al., 2022
Malaria	*Plasmodium berghei*	*Anopheles stephensi*	Microscopy, rapid diagnostic tests (RDTs)	Artificial neural networks (ANNs) coupled with matrix-assisted laser desorption/ionization time-of-fight (MALDI-TOF) mass spectrometry (MS)		Nabet et al., 2020
Rickettsial infection	*Rickettsia aeschlimannii*	*Haemaphysalis longicornis*	Immunofluorescence assay (IFA)	16S rRNA gene	Electron microscopy and liquid chromatography-mass spectrometry (LC-MS/MS)	Nawaz et al., 2020; Qi et al., 2022
American trypanosomiasis (Chagas disease)	*Trypanosoma cruzi*	*Triatoma dimidiata s.l.*	Microscopic examination	Genotype-by-sequencing (GBS), microscopy study, PCR and qPCR detection		Stevens et al., 2021
Schistosomiasis	*Schistosoma haematobium, Schistosoma japonicum*	Planorbis spp	Kato-Katz technique, circulating cathodic antigen (CCA) test, filtration technique and microscopic detection	Real-time PCR		Gaye et al., 2021
Zika	ZIKV	*Aedes aegypti* and *Aedes albopictus*	RT-PCR, NS1 antigen detection using ELISA, rapid point-of-care tests, IgM/IgG detection using EIA, IFA and rapid point-of-care test (Plaque-reduction neutralization test)	RT-PCR	Cryo-electron microscopy	Zhou et al., 2019; Parra et al., 2022
Japanese encephalitis	Japanese encephalitis virus (JEV)	*Culex tritaeniorhynchus*	IgM-capture ELISA	Cytochrome c oxidase subunit 1 (COI) bar-coding		Lessard et al., 2021
Chikungunya	CHIKV	*Aedes aegypti, Aedes albopictus* and *Culex quinquefasciatus*	RT–PCR	RT-qPCR		Cruz et al., 2020
Lymphatic filariasis	*Wuchereria bancrofti, Brugia malayi, Brugia timori.*	Anopheles spp, *Culex quinquefasciatus*, Aedes spp and Mansonia spp	Microscopic examination	qPCR	Nanoparticle tracking analysis and liquid chromatography-mass spectrometry (LC-MS/MS)	Harischandra et al., 2018; Entonu et al., 2020
Leishmaniasis	*Leishmania donovani*	*Phlebotomus argentipes*	Parasitological tests (microscopic examination, PCR) and serological tests (IFA, ELISA, Western blot)	PCR and sequencing method	Transmission electron microscope	Schnitzer et al., 2010; Wijerathna et al., 2021

**FIGURE 2:**
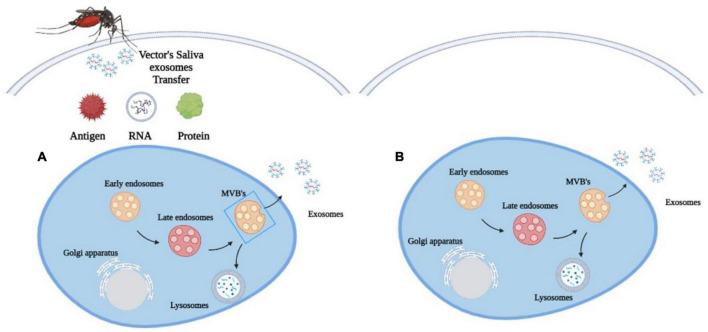
Exosomes biogenesis in **(A)** vector-borne diseases. Arthropods release exosomes into the host blood circulation through their saliva, which transfer antigens, proteins and RNA to modulate immune responses, gene expression and metabolic processes and **(B)** in other diseases. Exosomes are released only by the diseased cell.

When feeding on their hosts, arthropods release exosomes into the host blood through their saliva (Figure 2; Oliva Chávez et al., 2021). In response, the host cells will also release their own exosomes to inhibit the transmission of pathogens (Alenquer and Amorim, 2015). This increases exosome concentrations in the plasma and other body fluids (Opadokun and Rohrbach, 2021). As a result, exosomes from the host and pathogen will play distinct roles in the survival and suppression of vector-borne diseases.

The exosomes in the arthropod’s saliva can be considered a carrier for various biological processes in vector-borne diseases. They transfer antigens, proteins and RNA into the host cell to influence metabolic processes, gene expression and immunological responses (Figure 2; Yuan et al., 2021). Through this process, these exosomes may facilitate the transmission of arboviruses that cause dengue and encephalitis to mammals (Zhou et al., 2019). The discovery of exosomes produced by arthropods has spurred the development of new methods to prevent the spread of vector-borne diseases through the understanding of molecular determinants in the mode of pathogen transmission. A Malaysian study has found that the EV secreted by *Aedes albopictus* and *Aedes aegypti* contains a tetraspanin domain-containing glycoprotein known as Tsp29Fb that could facilitate the transmission of Dengue Virus 2 (DENV2) in host cells (Vora et al., 2018). This study has led to a novel proposal to restrict the transmission of DENV2 and other mosquito-borne flaviviruses to mammalian cells by inhibiting the effects of Tsp29Fb in mosquitoes using the GW4869 inhibitor. A proteomic study on exosomes of Drosophila cells infected by the cricket paralysis virus (CrPV) also revealed that they contained viral RNA to facilitate the spread of the pathogen’s genetic material in the host (Kerr et al., 2018).

Pathogen surveillance in arthropods may provide essential details for disease control. A central American study tried to discover the best method to survey the prevalence of *Trypanosoma cruzi* that causes Chagas disease in the vector *Triatoma dimidiata* (blood-sucking bug) (Stevens et al., 2021). The researchers concluded that using genotype-by-sequencing (GBS) was twice superior in detecting the pathogen in its vector compared with PCR assay (based on a test with the significant agreement; 53% vs. 19%) However, the difficulty of pathogen detection in insects may be caused by genetic variations in the parasite itself, and this can affect the disease surveillance and control process. Meanwhile, detection of the EV secreted by pathogens may also assist in the development of new clinical biomarkers and therapeutic agents. The study of exosomes in *Brugia malayi* has found that miRNA let-7 (lethal 7) could function as a biomarker for lymphatic filariasis disease (Zamanian et al., 2015). Table 3 summarizes the potential miRNA biomarkers for vector-borne diseases. Table 4 summarizes the exosomal miRNA biomarkers for diagnostic applications in vector-borne diseases.

**TABLE 3:** miRNA biomarker for vector-borne diseases.

Diseases	Sample	miRNA	Regulation	References
Malaria	Whole blood	hsa- miR-7977	Up-regulated	Kaur et al., 2018
Dengue	Serum	hsa-miR-21-5p, hsa-miR-590-5p, hsa-miR-188-5p, and hsa-miR-152-3p	Up-regulated	Ouyang et al., 2016
		hsa-miR-146a-5p	Down-regulated	
Schistosomiasis	Serum	miR-223		He et al., 2013
West Nile virus	Whole blood	miR-532-5p		Bavia et al., 2016
Zika virus	Mock cells	miR-145, miR-148a	Up-regulated	Castro et al., 2019
Chikungunya	Human skin fibroblast cells	miR15, miR-16, miR-17, let-7e, miR-125, miR-99, miR-23a		Parashar et al., 2018
West Nile virus	Human microglial cells	miR-3648, miR-3687, miR-129-5p, miR-572		Kumari et al., 2016
Japanese encephalitis	Human microglial cells	miR-3687 and miR-572	Up-regulated	Kumari et al., 2016
		miR-197-3p	Down-regulated	

**TABLE 4:** Exosomal miRNA expression in different vector-borne diseases.

Vector-borne diseases	Sample	Biomarkers	References
Malaria	Plasma exosomes	hsa-miR-150-5p and hsa-miR-15b-5p	Ketprasit et al., 2020
Schistosomiasis	Serum exosomes	miR-142-3p and miR-223-3p	Wu et al., 2020
Rickettsia	Endothelial exosomes	miR-23a and miR-30b	Liu et al., 2021
Chagas disease	Plasma exosomes	IFN- and IL-17	Madeira et al., 2021
Japanese encephalitis	Cerebrospinal fluid	miR-21-5p, miR-150-5p and miR-342-3p	Goswami et al., 2017

### Dengue

Dengue fever is a febrile illness caused by *Flavivirus* of the family *Flaviviridae.* This virus has a common size of between 40 and 65 nm and contains a positive single-stranded RNA genome (Murugesan and Manoharan, 2019). The WHO estimates its prevalence at 50–100 million cases each year in tropical countries (WHO Dengue, 2022). In the 21st century, dengue has emerged as one of the most significant mosquito-borne diseases that pose a severe threat to public health (Gurugama et al., 2010). Therefore, finding predictive indicators of severe infection will greatly aid in patient management and treatment allocations. Conventional diagnostic methods include the structural protein 1 (NS1) test, immunoglobulin G/M test and real-time PCR (Kabir et al., 2021). However, a comprehensive analysis of miRNAs circulating in the plasma of dengue patients has identified miR-122-5p as a potential biomarker for diagnosis and prognosis of the disease (Saini et al., 2020). Integrated bioinformatic analysis predicted that interferon-induced protein 44-like (IFI44L) and interferon-α inducible protein-6 (IFI6) could also be used as potential biomarkers of infection (Xie et al., 2021).

MiRNA expression may change during the different stages of infection, thus providing the potential biomarkers for treatment monitoring (Soe et al., 2018). Dengue virus infection causes platelet cells to release exosomes via the C-type lectin-like receptor 2 (CLEC2) signaling (Sung et al., 2019). Exosomes released from mosquito cells infected with dengue virus were observed to have an effect on native C6/36 cells, which shows that the exosomes played a role in virus dissemination (Reyes-Ruiz et al., 2019). Exosomes secreted from dengue virus infected cells has been observed to carry LC3-phosphatidylethanolamine conjugates (LC3 II), which is an autophagy marker (Wu et al., 2016). However, further knowledge on plasma exosomes and their exact function in the transmission dengue virus is still lacking.

### Malaria

Malaria is a vector-borne disease caused by plasmodium parasites carried by the female *Anopheles* mosquito (Walter and John, 2022). It is most prevalent in Africa, where 95% of the fatality occurs. In 2020 alone, it was estimated that there were about 241 million cases worldwide, with a death toll of 627,000 (WHO Malaria, 2022). To overcome this disease, an in-depth understanding of the parasite biology and mechanisms underlying the disease is urgently needed (Chen et al., 2021). Conventionally, microscopy or a rapid diagnostic kit is used to confirm the presence of malaria-causing parasites (CDC Malaria, 2022). Lately, studies have shown that plasma exosome also increases in malaria patients compared with healthy individuals (Nantakomol et al., 2011). These biomolecules play many important roles in intercellular communication during the infection process (Schorey et al., 2015). Exosomal plasma-derived hsa-miR-150-5p and hsa-miR-15b-5p have been studied for their potential use as disease biomarkers (Ketprasit et al., 2020). In the case of clinical management, malaria is difficult to treat due to the ability of the plasmodium parasite to develop drug resistance. One study has observed the production of biomolecules with potential function to pass on drug-resistance markers to susceptible parasites in gametocytogenesis stage, which is critical for disease transmission (Regev-Rudzki et al., 2013).

### Rickettsial infection

*Rickettsia* is a species of highly pleomorphic, non-motile, Gram-negative bacteria that may exist in coccus, bacilli and thread forms, and is transmitted via the bites of mites, fleas, lice, and ticks (Moreira et al., 2018). Being an obligate intracellular parasite, it has to infect eukaryotic host cells to survive and replicate, particularly endothelial cells. Even though rickettsial infections have similar clinical presentations, the causative species and epidemiology may differ regionally. Some examples include epidemic typhus (*Rickettsia prowazekii*, worldwide), the Australian tick typhus (*Rickettsia australis*, Australia), Rocky Mountain spotted fever (*Rickettsia rickettsii*, North America), rickettsialpox (*Rickettsia akari*, United States and Russia), oriental spotted fever (*Rickettsia japonica*, Japan) and African tick bite fever (*Rickettsia africae*, South Africa). To accurately diagnose and treat these diseases, it is crucial to understand both the typical symptoms and epidemiology of a specific area (Khamesipour et al., 2018). In severe rickettsiosis, endothelial barrier dysfunction occurs in the brain and lungs that results in edema. A recent study showed that endothelial exosomal miR-23a and miR-30b have potential role in targeting the endothelial barriers and may serve as an early diagnostic tool (Liu et al., 2021).

### Chagas disease

Chagas disease, also known as American trypanosomiasis, is a tropical parasitic disease caused by the protozoa *Trypanosoma cruzi*. It is transmitted mainly by bugs of the triatominae subfamily (also known as kissing bugs or vampire bugs) (Rassi et al., 2010). More than seven million people are infected with Chagas disease annually, mainly in Mexico, Central America and South America. It has been estimated to be responsible for 12,500 fatalities per year since 2006 (World Health Organization, 2022b). Chagas disease is diagnosed using microscopy and serological tests to detect trypomastigotes in blood (CDC Chagas, 2022). The significance of miR-208a levels in plasma as a potential biomarker for clinical prognosis of Chagas disease is illustrated in the chronic indeterminate phase, which is a progressive phase implicated in the development of chagas cardiomyopathy (Linhares-Lacerda et al., 2018). Plasma exosomes produced during disease infection may also induce different interferon (IFN) and Interleukin-17 (IL-17) expression, which may be used to track the development of the disease (Madeira et al., 2021).

### Schistosomiasis

Schistosomiasis is the second leading parasitic disease after malaria, which is caused by trematodes of the genus *Schistosoma* (Amoah et al., 2020). Schistosomiasis is prevalent in tropical and subtropical areas, with 236.6 million cases reported worldwide in 2019 (WHO Schistosomiasis, 2022). It can excrete certain proteins into the circulation of a final host (humans) to facilitate their parasitism (Liao et al., 2011). Currently, the diagnostic methods for this disease include stool and urine microscopy, besides serological testing for antischistosomal antibody (CDC Schistosomiasis, 2022). Different schistosome-specific miRNAs, like miR-10, miR-3479, miR-0001, and miR-10, have been discovered to describe the pathogen-specific short RNA community in the plasma of a final host infected with *Schistosoma japonicum* (Cheng et al., 2013). Currently, serum exosome-derived miR-142-3p and miR-223-3p are being used as diagnostic markers of schistosomiasis at an early stage (Patent number: CN110760589-A and CN110760590-A) (Wu et al., 2020). Some studies have shown that exosomal products can serve as prognostic and stage-predictive biomarkers of the disease (Meningher et al., 2017).

### Zika virus diseases

Zika virus (ZIKV) is a mosquito-borne virus transmitted by *Aedes* mosquitoes. It is closely related to dengue and other diseases related to viruses in the Flaviviridae family. The virus is enveloped and icosahedral, containing a positive sense single-stranded RNA as its genetic material (Marbán-Castro et al., 2021). Conventionally, ZIKV was detected in blood and other bodily fluids using RT-PCR; enzyme-linked immunosorbent assay (ELISA) for NS1 antigen; enzyme immunoassay (EIA) for IgM/IgG; immunofluorescence assay (IFA); and rapid point-of-care testing, such as the plaque-reduction neutralization test (PRNT) (World Health Organization, 2022c).

It is vital to differentiate ZIKV from other closely related flaviviruses that can cross-react when using conventional diagnostic methods like ELISA and EIA. And to this end, RT-PCR seems to be the most accurate tool. The detection of ZIKV in *Aedes aegypti and Aedes albopictus* mosquitoes using RT-PCR in São Paulo, Brazil, has helped to understand the transmission dynamics of ZIKV and potential risk of future outbreaks in several neighborhoods in the city (Parra et al., 2022). However, in the clinical setting, there is a need for a method that is fast and low cost. The EVs from the ZIKV-infected human saliva express typical EV biomarker tetraspanins CD9, CD63, and CD81 to prevent target cells (Conzelmann et al., 2020). The induction of miR-145 and miR-148a in postmortem brain samples from stillborn with severe congenital Zika syndrome cause neuronal dysfunction and malformation (Castro et al., 2019). These studies showed the role of extracellular vesicles and the miRNA in Zika infection.

### Japanese encephalitis

Japanese encephalitis virus (JEV) is a mosquito-borne disease transmitted by Culex mosquitoes. Like dengue, West Nile, Yellow Fever and Zika counterparts, it is part of the family Flaviviridae that causes viral encephalitis (Sharma et al., 2021). Conventionally, it may be diagnosed using ELISA, RT-PCR, and the plaque reduction neutralization test. However, there is still a need to develop more efficient, inexpensive, quicker, sensitive and time-saving techniques to detect JEV (Roberts and Gandhi, 2020). Exosome study in acute patients has found upregulated miRNAs like miR-21-5p, miR-150-5p, and miR-342-3p in the cerebrospinal fluid, which can be a potential diagnostic biomarker (Goswami et al., 2017). The host microRNA miR-34c-5p in JEV infection is downregulated and activates a notch pathway to modulate the microglia and mediate inflammation (Kumari et al., 2016). This showed that miRNA can regulate host response to JEV in microglial cells.

### Chikungunya

Chikungunya virus (CHIKV) is an alphavirus transmitted by *Aedes* and some *Culex* mosquitoes. It is a positive-sense, single-stranded RNA virus in the family Togaviridae that causes Chikungunya disease (Schwartz and Albert, 2010). Conventionally, Chikungunya is mainly diagnosed using RT-PCR. The study on extracellular vesicles released by CHIKV-infected cells contained the immunogenic components of virus as well as its genomic RNA. These EVs then helped other epithelial cells produce contagious virions (Le et al., 2022). Since there is a limited study on the role of exosomal miRNA in chikungunya, this study initiated to characterize extracellular vesicles in chikungunya infection.

### Lymphatic filariasis

Lymphatic filariasis (LF) is a mosquito-borne tropical disease caused by parasitic nematodes, such as *Wuchereria bancrofti, Brugia malayi*, and *Brugia timori* (Lourens and Ferrell, 2019). These parasites can modulate the host immune response. However, there is still a lack of knowledge to understand this mechanism. In lymphatic filariasis, the first study in parasite *B. malayi* exosome reported the mechanism of host response on parasite infection. This study also suggested that the further investigation in extracellular vesicles may led to the discovery of novel treatment approaches and diagnostic biomarkers for lymphatic filariasis infection (Zamanian et al., 2015). The parasite-derived EV study on filarial nematode *B. malayi* has suggested new screening platforms for anti-filarial drug development (Harischandra et al., 2018).

### Leishmaniasis

Leishmaniasis is a vector-borne disease transmitted by the protozoan parasites infected adult female phlebotomine sand flies. It is caused by the leishmania parasite that belongs to the family Trypanosomatidae. The conventional methods to diagnose leishmaniasis are parasitological tests (microscopic examination, PCR detection) and serological tests, such as an indirect IFA, ELISA, and Western blot (WHO Leishmaniasis, 2023). The exosomal miRNA study in eukaryotic Leishmania infection cell reported that the exosomal miR122 can interfere with lipoprotein status in the host cell after hepatic dysfunctions induced by the parasite (Di Loria et al., 2020). This study showed that exosomal miRNA from the vector can involve in the host metabolism modulation. The parasite detection of *Leishmania donovani* in arthropod *Phlebotomus argentipes* using DNA amplification (PCR) and sequencing methods help to study the disease epidemiology in Kurunegala District, Sri Lanka (Wijerathna et al., 2021). The parasite Leishmania-infected dendritic cells release the exosomes to protect against the parasite infection. It provokes new knowledge to develop a cell-free vaccine for immune prophylaxis against leishmaniasis (Schnitzer et al., 2010).

## Conclusion

The currently available diagnostic methods for many vector-borne diseases are insufficient and have many limitations. Most of the detection is dependent on the existence of signs and symptoms, or antibodies of particular pathogen. Therefore, there is an urgent need to support the development of new biomarker techniques in diagnostic platforms. Plasma-derived exosome miRNAs are more stable, reliable and highly concentrated than any other bodily fluids. Recent studies have indicated that exosomal miRNA within excretory-secretory products may serve as a route for successfully mediating parasite-host contacts for host manipulation. Interestingly, previous studies discovered certain differentially expressed host plasma-derived exosome miRNAs that may be potential diagnostic and prognostic biomarker of vector-borne diseases. However, there is still limited research of plasma exosome in vector-borne diseases as compared with other diseases such as malignancy. The lack of consistent definitions and a standard method causes considerable confusion in the laboratory study of exosomes. We believe that further research in this area will solve the problem, and provide the new ideas and approaches for the application of plasma-derived exosome miRNA in clinical practice.

## Author contributions

GV conducted the literature search and drafted the manuscript. WS conceived the idea of this review and gathered the data. WNS, SI, and AM gave critical ideas in drafting the manuscript. All authors approved the final submission of this manuscript.
